# Combination Therapy of an Intestine-Specific Inhibitor of Microsomal Triglyceride Transfer Protein and Peroxisome Proliferator-Activated Receptor *****γ***** Agonist in Diabetic Rat

**DOI:** 10.1155/2014/890639

**Published:** 2014-03-17

**Authors:** Shohei Sakata, Yasuko Mera, Yukiharu Kuroki, Reiko Nashida, Makoto Kakutani, Takeshi Ohta

**Affiliations:** Biological/Pharmacological Research Laboratories, Central Pharmaceutical Research Institute, Japan Tobacco Inc., 1-1 Murasaki-cho, Takatsuki, Osaka 569-1125, Japan

## Abstract

We investigated effects on glucose and lipid metabolism in combination of JTT-130, a novel intestine-specific microsomal triglyceride transfer protein (MTP) inhibitor, and pioglitazone, peroxisome proliferator-activated receptor (PPAR) **γ** agonist. Male Zucker diabetic fatty rats were divided into 4 groups: control group, JTT-130 treatment group, pioglitazone treatment group, and combination group. The Zucker diabetic fatty rats were fed a regular powdered diet with JTT-130 and/or pioglitazone as a food admixture for 6 weeks. Effects on glucose and lipid metabolism were compared mainly between JTT-130 treatment group and combination group. JTT-130 treatment showed good glycemic control, while the plasma glucose and glycated hemoglobin levels in combination group were significantly decreased as compared with those JTT-130 treatment group. The reduction in the plasma triglyceride and free fatty acid levels in combination group was higher than that in JTT-130 treatment group, and glucose utilization was significantly elevated in adipose tissues. In Zucker diabetic fatty rats, combination treatment of JTT-130 and pioglitazone showed better glycemic control and a strong hypolipidemic action with an enhancement of insulin sensitivity. Combination therapy of MTP inhibitor and PPAR**γ** agonist might be more useful in the treatment of type 2 diabetes accompanied with obesity and insulin resistance.

## 1. Introduction

Diabetes mellitus is not a single disease but a group of metabolic disorders affecting a huge number of populations worldwide [1, 2]. It is mainly characterized by chronic hyperglycemia, resulting from defects in insulin secretion and insulin action. With progress in elucidation of diabetic etiology, oral hypoglycemic drugs, such as sulfonylureas, *α*-glucosidase inhibitor, thiazolidinediones, and dipeptidyl-peptidase (DPP) IV inhibitor, have been successfully developed so far. Recently, moreover, combination therapies have become important in diabetic treatment [3, 4]. It seems useful to combine different antidiabetic agents based on specific needs and contraindications in an individual patient. Addressing not only glycemic control but also the underlying pathophysiological etiology might help to improve the prognosis in type 2 diabetic patients in the long run [5, 6]. Beyond lowering blood glucose levels, each combination of different antidiabetic drugs evolves specific pleiotropic effects, which might be considered on an individual basis in certain patients. Treatment with pioglitazone in type 2 diabetes was shown to improve insulin resistance and blood glucose levels without increasing the risk of hypoglycemia. Pioglitazone is approved in combination with several other antidiabetic drugs for treatment of type 2 diabetes [7, 8].

JTT-130, a novel intestine-specific inhibitor of microsomal triglyceride transfer protein (MTP), suppresses the absorption of dietary fat and cholesterol in the intestine and decreases plasma triglyceride and total cholesterol levels without accumulation of hepatic triglyceride [9, 10]. JTT-130 suppresses high-fat diet-induced obesity and improves glucose and lipid metabolic abnormalities with the elevation of plasma glucagon-like peptide-1 (GLP-1) levels in SD rats and Zucker diabetic fatty rats [11–13]. In this study, we investigated the effects on glucose and lipid metabolism in combination treatment of JTT-130 and pioglitazone in Zucker diabetic fatty rats.

## 2. Materials and Methods

### 2.1. Materials

JTT-130,   diethyl-2-({3-dimethylcarbamoyl-4-[(4′-trifluoromethylbiphenyl-2-carbonyl) amino] phenyl}acetyloxymethyl)-2-phenylmalonate, and pioglitazone were synthesized by Japan Tobacco Inc. (Osaka, Japan). All other reagents used in this study were obtained commercially.

### 2.2. Animals and Diets

Male Zucker diabetic fatty rats were obtained from Charles River Laboratories (Yokohama, Japan), individually housed with controlled temperature, humidity, and lighting (23 ± 3°C, 55 ± 15%, and a 12 h light/dark cycle with lights on at 8:00 AM), and provided with a powder diet (CRF-1; Oriental Yeast, Osaka, Japan) and water* ad libitum*. Zucker diabetic fatty rats were divided into 4 groups: a control group, JTT-130 treatment group, pioglitazone treatment group, and combination group. The assignment of rats was performed by body weight and nonfasting plasma parameters. Zucker diabetic fatty rats in the JTT-130 treatment group were fed a powder diet mixed with an appropriate amount of JTT-130 (0.01-0.02%) to achieve a daily dose of approximately 10 mg/kg for 42 days, from 7 to 13 weeks of age. The rats in the pioglitazone treatment group were fed a powder diet mixed with an appropriate amount of pioglitazone (0.001-0.002%) to achieve a daily dose of approximately 0.3 mg/kg. The rats in the combination group were fed a powder diet mixed with JTT-130 (0.01-0.02%) and pioglitazone (0.001-0.002%). Food intake and body weight were measured every 3 or 4 days during the experimental period. All procedures were conducted in accordance with the guidelines of the Japan Tobacco Animal Care Committee.

### 2.3. Measurement of Blood Chemical Parameters

Nonfasting plasma parameters, such as glucose, glycated hemoglobin, insulin, triglyceride, total cholesterol, and free fatty acid levels, were examined every 7 days. Blood samples were collected from the tail vein. Glucose, glycated hemoglobin, triglyceride, and total cholesterol levels were measured using commercial kits (Roche Diagnostics, Basel, Switzerland) and an automatic analyzer (Hitachi7170S, Tokyo, Japan). Plasma insulin level was measured with a rat-insulin enzyme-linked immunosorbent assay kit (Morinaga Institute of Biological Science, Yokohama, Japan). Plasma free fatty acid level was measured using NEFA* C*-test (Wako Pure Chemicals Industries Ltd., Osaka, Japan).

### 2.4. Glucose Utilization in Adipose Tissues

Small pieces (approximately 200 mg) of epididymal and mesenteric adipose tissues were incubated in Hank's balanced salt solution (pH 7.4) containing D-[U-^14^C]-glucose (GE Healthcare UK, Little Chalfont, Buckinghamshire, England) in the absence or presence (100 nmol/L) of insulin at 37°C for 2 h. After stopping the reaction by the addition of 0.05 mol/L H_2_SO_4_, the produced ^14^CO_2_ was trapped with filter paper. Radioactivity of filter paper was measured using a liquid scintillation counter (TRI-CARB 2500TR, Packard BioScience, Waltham, MA, USA). The protein contents of the adipose tissue pieces were determined using a BCA protein assay kit (Piece Biotechnology, Rockford, IL, USA), and the radioactivity of filter paper was normalized for comparison with the tissue protein content.

### 2.5. Statistical Analysis

Data were expressed as mean values ± standard deviation or + standard deviation. Tukey or Steel-Dwass test was used to determine statistical significance.* P* < 0.05 was considered statistically significant.

## 3. Results

### 3.1. Food Intake and Body Weights

**Figure 1:**
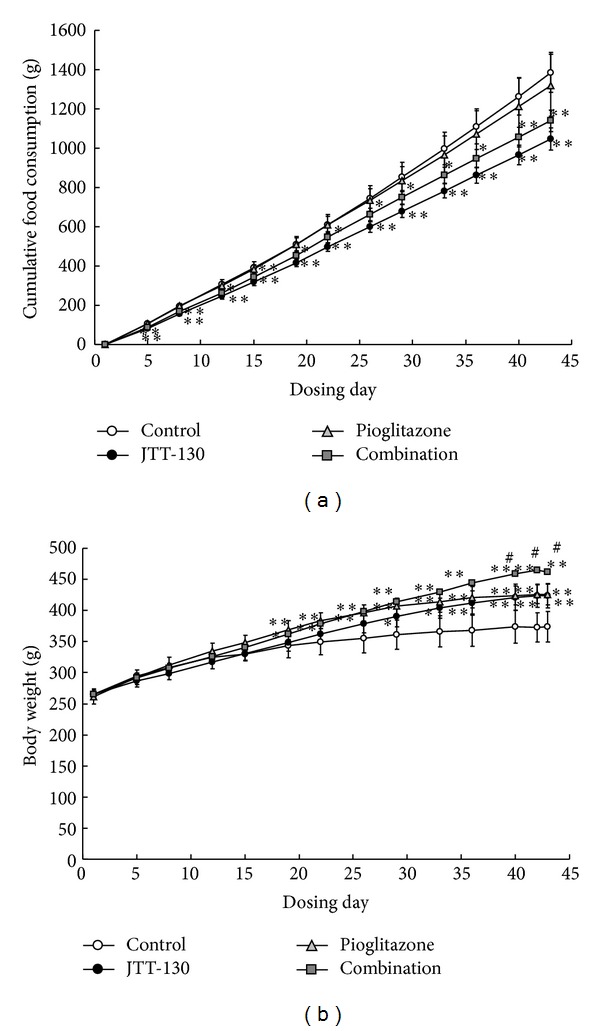
Changes in food intake (a) and body weight (b) in control group, JTT-130 treatment group, pioglitazone treatment group, and combination group. Data represent mean values ± standard deviation (*n* = 6). *P < 0.05, **P < 0.01, significantly different from control group. ^#^ *P* < 0.05, significantly different from JTT-130 treatment group.

Changes in cumulative food intake and body weight are shown in Figure 1. The cumulative food intakes were decreased in JTT-130 treatment and combination groups after day 4 of treatment as compared with those in control group, but there were no significant differences in the food intake between the groups (Figure 1(a)). The cumulative food intakes in pioglitazone group were comparable to those in control group during the experimental period.

The body weights in JTT-130 treatment group were slightly lower in the early period of experiment and, inversely, increased after day 33 of treatment as compared with those in control group, and the body weights in pioglitazone treatment and combination groups were increased after day 19 (Figure 1(b)). The body weights in combination group were higher than those in JTT-130 treatment group after day 40 of treatment.

### 3.2. Blood Chemical Parameters

**Figure 2:**
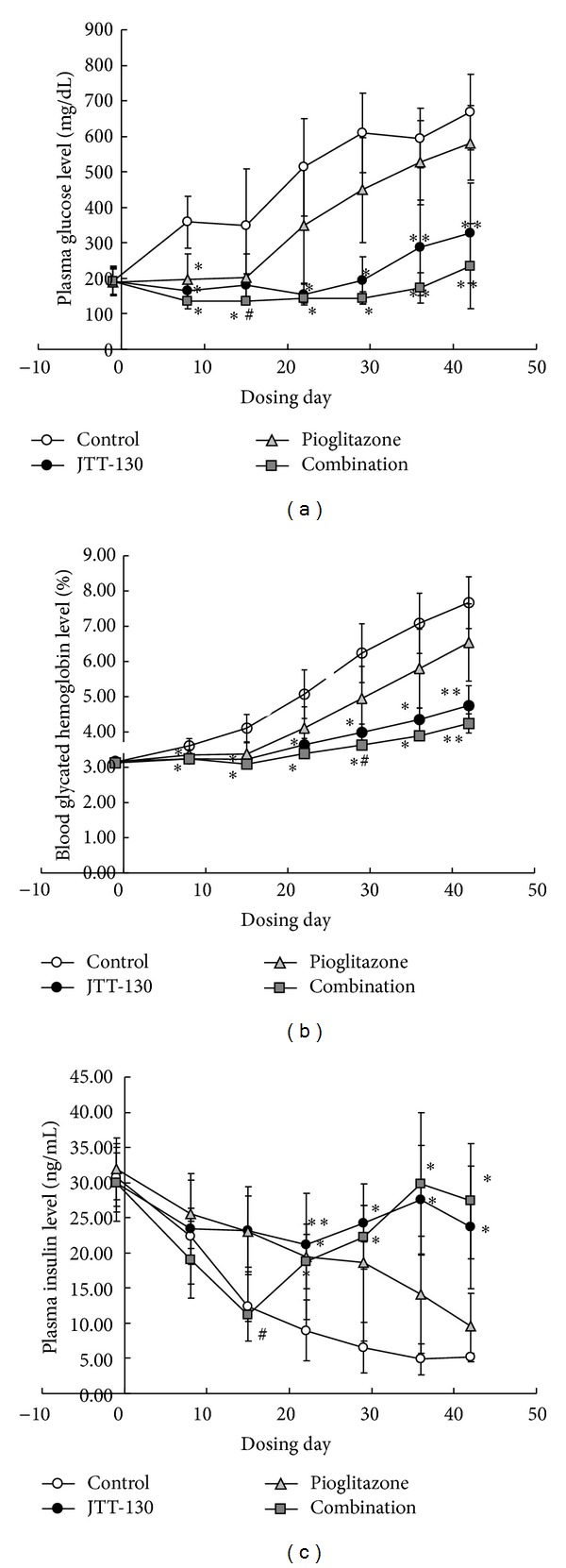
Changes in blood glucose (a), glycated hemoglobin (b), and insulin (c) levels in control group, JTT-130 treatment group, pioglitazone treatment group, and combination group. Data represent mean values ± standard deviation (*n* = 6). *P < 0.05, **P < 0.01, significantly different from control group. ^#^ *P* < 0.05, significantly different from JTT-130 treatment group.

**Figure 3:**
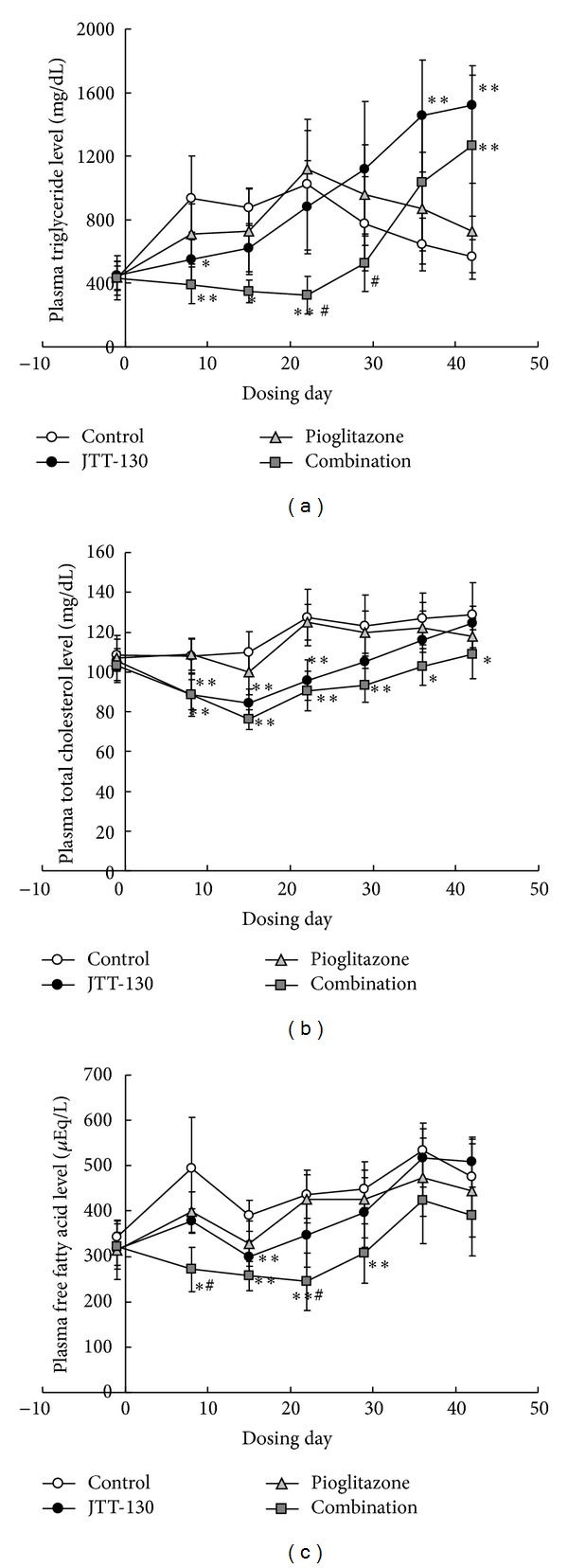
Changes in blood triglyceride (a), total cholesterol (b), and free fatty acid (c) levels in control group, JTT-130 treatment group, pioglitazone treatment group, and combination group. Data represent mean values ± standard deviation (*n* = 6). *P < 0.05, **P < 0.01, significantly different from control group. ^#^ *P* < 0.05, significantly different from JTT-130 treatment group.

Changes in blood chemical parameters are shown in Figures 2 and 3. Zucker diabetic fatty rats in control group showed severe hyperglycemia with the significant elevation in the plasma glucose and glycated hemoglobin levels with time. The plasma glucose levels in JTT-130 treatment and combination groups were significantly decreased after day 7 of treatment as compared with control group. The plasma glucose levels in pioglitazone treatment group were also lower than those in treatment group, but the effects were significant only at day 7. Moreover, the plasma glucose levels in combination group were significantly decreased at day 14 of treatment, as compared with those in JTT-130 treatment group (control group, 359 ± 159 mg/dL; JTT-130 treatment group, 182 ± 32 mg/dL; combination group, 137 ± 23 mg/dL) (Figure 2(a)). Along with the plasma glucose levels, the glycated hemoglobin levels in JTT-130 treatment and combination groups were significantly decreased after day 7. Furthermore, the glycated hemoglobin levels in combination group were significantly decreased at day 28 of treatment, as compared with those in JTT-130 treatment group (control group, 4.11 ± 0.39%; JTT-130 treatment group, 3.22 ± 0.12%; combination group, 3.09 ± 0.10%) (Figure 2(b)). The glycated hemoglobin levels in pioglitazone treatment group tended to be decreased as compared with those in control group but not significantly. The plasma insulin levels in JTT-130 treatment and combination groups were significantly increased after day 21 of treatment as compared with those in control group, and the plasma insulin levels in pioglitazone group were significantly increased only at day 21. At day 14 of treatment, the plasma insulin levels in combination group showed a significant reduction as compared with those in JTT-130 treatment group (Figure 2(c)).

The plasma triglyceride levels in JTT-130 treatment group were lower in early period of experiment and significantly decreased on day 7 of treatment as compared with that in control group, but the levels were, inversely, elevated after day 35. The plasma triglyceride levels in combination group were also lower and significantly decreased from day 7 to 21 of treatment, as compared with those in control group, but the levels were also, inversely, increased at day 42 (Figure 3(a)). The plasma triglyceride levels in combination group showed significant reduction as compared with those in JTT-130 treatment group at days 21 and 28 of treatment (control group, 1021 ± 412 mg/dL; JTT-130 treatment group, 881 ± 293 mg/dL; combination group, 327 ± 117 mg/dL, at day 21) (Figure 3(a)). The triglyceride levels in pioglitazone treatment group were comparable to those in control group during the experimental period. As compared with control group, the plasma total cholesterol levels in JTT-130 treatment group were significantly decreased from day 7 to 21 of treatment and the levels in combination group were significantly decreased after day 7 of treatment (Figure 3(b)). The total cholesterol levels in pioglitazone treatment group were comparable to those in control group. As compared with control group, the plasma free fatty acid levels in JTT-130 treatment group were significantly decreased at day 14, and the levels in combination group were decreased from day 7 to 28 of treatment. The plasma free fatty acid levels in combination group showed significant reduction as compared with those in JTT-130 treatment group (control group, 437 ± 53 *μ*Eq/L; JTT-130 treatment group, 347 ± 71 *μ*Eq/L; combination group, 244 ± 63 *μ*Eq/L, at day 21) (Figure 3(c)). There were no significant differences in the free fatty acid levels between pioglitazone group and control group.

### 3.3. Glucose Utilization in Adipose Tissues

**Figure 4:**
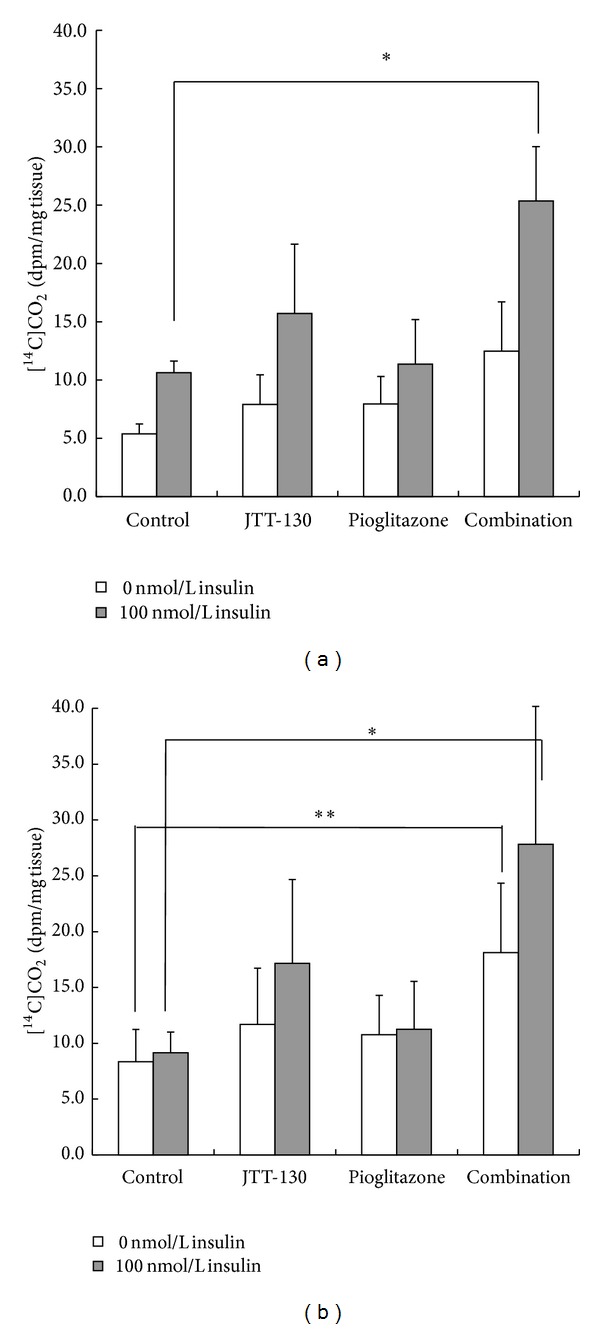
Effect on glucose utilization in the adipose tissues of epididymal fat (a) and mesenteric fat (b) in control group, JTT-130 treatment group, pioglitazone treatment group, and combination group. Data represent mean values + standard deviation (*n* = 6). *P < 0.05, **P < 0.01, significantly different from control group.

To examine the effects of JTT-130 on glucose metabolism in the adipose tissues, we evaluated the abilities of epididymal and mesenteric adipose tissues to metabolize glucose to carbon dioxide using D-[U-^14^C]-glucose. The production of carbon dioxide in the adipose tissues in combination group was significantly higher than that in control group in both the epididymal and mesenteric fat (Figure 4). The production of carbon dioxide in JTT-130 treatment and pioglitazone groups tended to be increased but not significantly.

## 4. Discussion

Type 2 diabetes mellitus is a progressive disease characterized by an impairment of insulin action and failure of pancreatic *β*-cells to compensate for the enhanced insulin demand. Several oral diabetic agents, with actions such as enhancement of insulin sensitivity in insulin target organ and insulin secretion from pancreas, have been approved for type 2 diabetes management so far. Combination therapies using these antidiabetic drugs have been employed in recent years.

JTT-130, a novel intestine-specific MTP inhibitor, improves the glucose and lipid metabolic abnormalities in obese diabetic models without any hepatotoxicity [11–13]. JTT-130 was shown to ameliorate impaired glucose metabolism in high-fat diet-induced obese rats and Zucker diabetic fatty rats with reduced food intake and body weight gain and thus is expected to be useful as a drug for the treatment of type 2 diabetes accompanied with obesity-related metabolic disorders. Pioglitazone, peroxisome proliferator-activated receptor (PPAR)**γ** agonist, is an insulin-sensitizing agent available for treatment of type 2 diabetes. The mechanism of action involves binding to PPAR**γ**, a transcription factor that regulates the expression of specific genes, especially in fat cells [14]. Pioglitazone has been shown to interfere with expression and release of mediators of insulin resistance originating from adipose tissue. In this study, we investigated the combination effect of pioglitazone on JTT-130 treatment. Since significant pharmacological effects on 10 mg/kg of JTT-130 and 0.3 mg/kg of pioglitazone were confirmed in our preliminary and previous studies [12], those doses were established in the present study.

The glucose-lowering effect in combination group was more significant than that in JTT-130 treatment group (Figure 2(a)), and the glycated hemoglobin levels in combination group showed lower levels than in JTT-130 treatment group (Figure 2(b)). The value of glycated hemoglobin in ZDF rats (approximately 4% at 11 weeks of age) was lower than that of human, but similar values in diabetic rats were reported in other studies [12, 15]. In brief, better glucose control was achieved by combination of pioglitazone with JTT-130 treatment. The good glycemic control was considered to be caused by the interaction between an improvement of glucose metabolism by feeding restriction with JTT-130 and an enhancement of insulin sensitivity with pioglitazone. The plasma insulin levels at day 14 in combination group were significantly decreased as compared with those in JTT-130 treatment group (Figure 2(c)), suggesting that the combination treatment might enhance the insulin sensitivity more than JTT-130 alone. Moreover, the inhibition of progression of diabetes in JTT-130 and combination groups might induce the increases of body weight and blood insulin level after day 20 of treatment (Figures 1(b) and 2(c)).

Combination treatment significantly decreased the plasma triglyceride and free fatty acid levels (Figures 3(a) and 3(c)). These results suggested that the combination treatment ameliorated impaired lipid metabolism via the suppressed fat absorption by JTT-130 and the enhanced insulin sensitivity by pioglitazone, and therefore reduction of lipotoxicity in the combination treatment group was expected. Thus, the good glycemic control achieved in the combination treatment group might be caused not only by enhanced insulin sensitivity or decreased food intake but also the reduction in lipotoxicity. The current study using D-[U-^14^C]-glucose showed that combination treatment of JTT-130 and pioglitazone significantly increased glucose utilization as compared with the monotherapy in adipose tissue, indicating that the combination therapy enhanced the insulin sensitivity (Figure 4).

We have previously reported that JTT-130 improved glucose intolerance with increase in the plasma GLP-1 levels in high-fat diet-induced obese rats [13], and thus the elevated GLP-1 might contribute the combination effects in the current study. In fact, there have been some reports on the combination therapies of incretin-related drugs and insulin sensitizers. In combination study using Zucker diabetic fatty rats with liraglutide, a human GLP-1 analogue, and ragaglitazar, a dual PPAR *α*/**γ** agonist, nonfasting blood glucose level was significantly decreased in combination therapy after treatment for 1-2 weeks, and there was a significant interaction between combination therapy and monotherapy for glycated hemoglobin level after 30-31 days of treatment [16]. Additionally, in combination study using ob/ob mice with alogliptin, a DPP-4 inhibitor, and pioglitazone, the combination therapy improved glycemic control and lipid profiles; moreover, increased pancreatic insulin contents were shown in the study [17]. Those combinations were shown to be useful also in a clinical trial [18].

In further study, the mechanism of synergistic effects between JTT-130 and pioglitazone such as protection of pancreas and preventive action of ectopic fat accumulation should be investigated, and merits of the combination therapy elucidated. Additionally, it is of importance for development of JTT-130 to elucidate the synergistic effect with pioglitazone treatment observed in this study, since pioglitazone has been widely approved as an antidiabetic agent.

In summary, combination treatment of JTT-130 and pioglitazone showed good glycemic control and a strong hypolipidemic action with an enhancement of insulin sensitivity in Zucker diabetic fatty rats. The combination therapy might be useful in the treatment of type 2 diabetes accompanied by insulin resistance.
